# Reduced Self-Awareness Following a Combined Polar and Paramedian Bilateral Thalamic Infarction. A Possible Relationship With SARS-CoV-2 Risk of Contagion?

**DOI:** 10.3389/fpsyg.2020.570160

**Published:** 2020-10-02

**Authors:** Massimo Bartoli, Sara Palermo, Mario Stanziano, Giuseppina E. Cipriani, Daniela Leotta, Maria C. Valentini, Martina Amanzio

**Affiliations:** ^1^Department of Psychology, University of Turin, Turin, Italy; ^2^European Innovation Partnership on Active and Healthy Ageing, Brussels, Belgium; ^3^Neuroradiology Unit, Fondazione IRCCS Istituto Neurologico “Carlo Besta”, Milan, Italy; ^4^Postgraduate School of Radiodiagnostics, University of Milan, Milan, Italy; ^5^Martini Hospital, Neurology Unit, Turin, Italy; ^6^Neuroradiology Unit, Azienda Ospedaliera Universitaria “Città della Salute e della Scienza di Torino”, Turin, Italy

**Keywords:** polar and paramedian thalamic infarction, reduced self-awareness, executive functions, SARS-CoV-2, case study

## Abstract

Reduced self-awareness is a well-known phenomenon investigated in patients with vascular disease; however, its impact on neuropsychological functions remains to be clarified. Importantly, selective vascular lesions provide an opportunity to investigate the key neuropsychological features of reduced self-awareness in neurocognitive disorders. Because of its rarity, we present an unusual case of a woman affected by a combined polar and paramedian bilateral thalamic infarction. The patient underwent an extensive neuropsychological evaluation to assess cognitive, behavioral, and functional domains, with a focus on executive functions. She was assessed clinically in the acute phase and after 6 months from the stroke, both clinically and by magnetic resonance imaging. The patient developed a cognitive impairment, characterised by prevalent executive dysfunction associated with reduced self-awareness and mood changes, in terms of apathy and depression. Such condition persisted after 6 months. In May 2020, the patient underwent the serology test in chemiluminescence to detect IgG antibodies against SARS-CoV-2. The result of the quantitative test highlighted a high probability of previous contact with the virus. We suggest that reduced self-awareness related to executive dysfunction and behavioral changes may be due to combined polar and paramedian bilateral thalamic lesion. Metacognitive–executive dysfunction affecting the instrumental abilities of everyday life might make people less able to take appropriate precautions, facilitating the risk of SARS-CoV-2 contagion.

## Introduction

Awareness of illness is the more general theoretical term used to describe the ability to detect, distinguish, and diagnose the occurrence of different deficits in cognitive and affective domains (Amanzio et al., 2011).

Considering acquired brain injury (ABI), reduced self-awareness is a phenomenon characterised by impairments in recognising deficits together with their impact on the patient’s functioning and, consequently, in making realistic plans (Palermo et al., 2014).

Studies on reduced self-awareness in brain injury patients showed the key role played by the frontal lobes (Bach and David, 2006; Ownsworth et al., 2007) and subcortical regions (Starkstein et al., 1992). In particular, specific cingulofrontal areas dysfunctions (Palermo et al., 2014), damages involving primarily the temporoparietal junction (Devinsky, 2008), the lateral ventricles, the frontal horns, and diencephalic regions (Starkstein et al., 1992)—such as the thalamus and the basal ganglia (Starkstein et al., 2010)—might contribute to a reduced self-awareness (Devinsky, 2008; Starkstein et al., 2010; Palermo et al., 2014; Bourlon et al., 2017), also in terms of interoceptive awareness (i.e., the perception of heartbeat, breathing, hunger, thirst, and visceral sensations) (Raimo et al., 2019). De Witte et al. (2011) confirmed that the thalamus might be considered an attractive “hub” for the study of reduced self-awareness of cognitive deficits in ABI. Those authors reviewed 465 patients with vascular thalamic lesions, finding that two thirds of those with bilateral thalamic damage presented specific cognitive and behavioral deficits, such as reduced self-awareness and executive dysfunction, disrupted memory, constructional apraxia, disorientation, and global cognitive deficits associated with behavioral abnormalities (De Witte et al., 2011). Stroke in the left dorsomedian thalamus (Lanna et al., 2012) and bilateral paramedian thalamus (Rusconi et al., 2014) had been previously associated with anosognosia.

Regarding the association between executive dysfunction and reduced self-awareness, frontal areas seem to be implicated in self-awareness and in the control of cognitive functioning. Thus, in ABI patients, a reduction of awareness might be considered as a damage in self-monitoring (Stuss and Benson, 1984). This mechanism also occurs in patients with neurodegenerative disorders, such as frontotemporal dementia (FTD) (O’Keeffe et al., 2007; Amanzio et al., 2016; Levy et al., 2018), Alzheimer (Amanzio et al., 2010, 2011, 2013) and Parkinson (Amanzio et al., 2014; Palermo et al., 2017) diseases. Moreover, deficits in basic executive functions, i.e., cognitive set-shifting, response inhibition, and self-monitoring, have been previously proposed as possible mechanisms of reduced self-awareness in patients with a selective anterior cingulate cortex ABI (Palermo et al., 2014).

The present report illustrates the unusual case of a patient with self-awareness reduction due to combined polar and paramedian bilateral thalamic infarction.

The aim of this study is to outline the association among bilateral thalamic stroke, reduced self-awareness, and executive dysfunction. Such aspects have not been evaluated in the literature on patients with thalamic lesions yet. We would also suggest that reduced awareness related to executive dysfunction and consequent deficits in the instrumental activities of daily living (iADL) may be considered in assessing the possibility of SARS-CoV-2 contagion risk, as they would affect the subject’s ability to take appropriate precautions.

## Case Presentation: Neurological and Clinical Evaluation

At the time of this study, G.A. was a 63-year-old married woman, with 5 years’ education. She had normal developmental milestones and no medical history of note. She had been a factory worker all her life, but at the time of evaluation, she retired. Her father died at an early age due to unspecified causes, her mother died at 62 for complications of surgery. She had five brothers and two sisters, all deceased (two of which at a young age, one for stroke and one for cancer).

Her presenting symptoms were drowsiness, loss of balance, and strength, which have suddenly developed within few hours. Then, G.A. lost consciousness for a short while. She was transferred to the Emergency Department at the “Martini” Hospital in Turin, where she received adequate assistance.

At admission, her level of consciousness was fluctuating; no sensory-motor deficits were observed, pupils were normal and cranial nerves examination was unremarkable. The score on the National Institute of Health Stroke Scale (Brott et al., 1989) was 3 (minor stroke).

Brain computed tomography (CT) showed bilateral thalamic hypodensity suggestive of bilateral infarction; angio-CT showed no occlusion of large vessels. Electrocardiogram disclosed atrial fibrillation, which highlighted the cardioembolic nature of stroke. Thrombolysis was not considered because the onset of symptoms was >4.5 h. Laboratory analyses were unremarkable, and vital signs were normal, except for mild hypertension.

Subsequently, G.A. was admitted to the neurological unit and subjected to more in-depth neurological investigations. The patient showed a slowly progressive and spontaneous amelioration of vigilance and consciousness. Five days after the admission, she could walk independently and appeared quite alert with reduced time and space orientation. Her speech was hypophonic and slowed down, although she spoke correctly. She presented quite apathetic and depressed.

Specifically, the neurologist conducted an interview with the patient and her primary caregiver (the cohabitant husband) before the neuropsychological assessment, carried out five days after admission to the neurological department (T0). The interview allowed collecting patient’s information, double checked with the primary caregiver, through the following topics: demographic data and marital status, daily living habits, and remote anamnesis that excluded family history of neurodegenerative diseases. Finally, functional anamnesis was carried out in order to investigate aspects useful to set up an accurate neuropsychological assessment.

After spending 2 weeks in hospital, G.A. left the neurology unit and went home with a therapy consisting of oral anticoagulation therapy and folic acid. Neurologists recommended a neuropsychological rehabilitation focused on cognitive affected domains. Finally, the patient was asked to perform a follow-up examination after 6 months.

**FIGURE 1:**
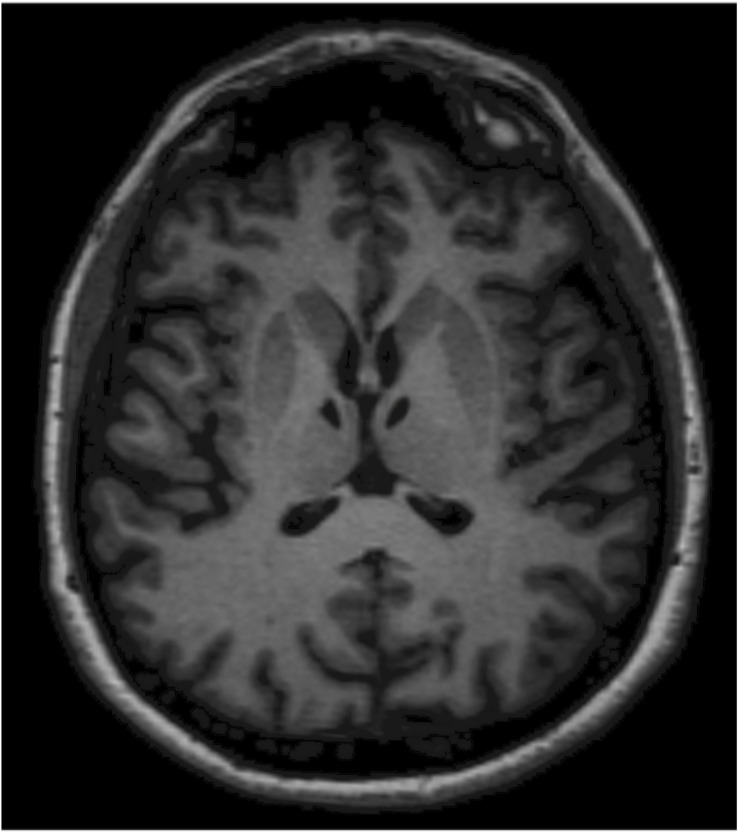
Magnetic resonance imaging (MRI) shows lacunar thalamic infarcts symmetrically located within the polar and paramedian vascular territories of both thalami. Lesions are well-demarcated and isointense to cerebrospinal fluid as observed in chronic settings. The anatomical symmetry was the distinctive feature in this patient.

At the follow-up examination, 6 months later (T1), during the neurological evaluation, G.A. appeared alert and collaborative, adequate on relationship. Her speech was fluent and correct. Her ideation was sometimes slowed down, but always consistent with the themes proposed by the examiner. The neurological exam disclosed neither nystagmus nor other cranial nerve alterations. G.A. maintained Mingazzini I and II for more than 60 s. All tests on segmental cerebellar were accurate. She showed symmetrical tendon reflexes. G.A. ambulated independently and presented a good motor and functional recovery. At T1, G.A. also underwent a magnetic resonance imaging (MRI), which showed combined polar and paramedian bilateral thalamic infarction (Figure 1).

Subsequently some of the patients were contacted, through their caregivers, during and after the lockdown period, to make sure of their state of health and of SARS-CoV-2 risk of contagion. In particular, in May 2020, G.A. underwent the serology test in chemiluminescence to detect IgG antibodies against SARS-CoV-2, recommended by the Italian Ministry of Health (Ministero della Salute, 2020).

### Neuropsychological Evaluation

G.A. was assessed during the acute phase (T0) and after 6 months (T1), undergoing the same neuropsychological evaluation, to estimate possible changes in response to the administrated therapy.

Her premorbid intellectual efficiency was assessed with the Brief Intelligence Test (TIB: Colombo et al., 2002), which is the Italian version of the National Adult Reading Test (Nelson, 1982). Her global cognition was measured with the Addenbrooke’s Cognitive Examination–Revised (ACE-R: Mioshi et al., 2006), which includes the Mini-Mental State Examination (MMSE) score (Folstein et al., 1975).

A detailed neuropsychological evaluation of specific cognitive domains were performed, considering: memory (Corsi Test; Digit Span; Incidental Semantic Memory; Rey Memory Test; Breve Racconto; Spinnler and Tognoni, 1987); language (Token Test: De Renzi and Vignolo, 1962; semantic verbal fluency: Spinnler and Tognoni, 1987); visuoconstructive abilities and praxia (Coping Design: Gainotti et al., 1977; buccofacial and ideomotor Apraxia Test: Spinnler and Tognoni, 1987); and deductive ability (Coloured Progressive Matrices-36: Spinnler and Tognoni, 1987). In order to exclude unilateral visual–attentional neglect, space, and percepts exploration deficits, the following tests were performed: the Bells Test (Gauthier et al., 1989), lines bisection and cancellations test (Spinnler and Tognoni, 1987), and the entangled figures test (Mondini et al., 2011). Executive functions and attention were measured using: the Montreal Cognitive Assessment (MoCA: Schweizer et al., 2012), the Attentional Matrices test (Spinnler and Tognoni, 1987), the Trial Making Test part A, B (TMT: Reitan and Wolfson, 1994), Phonemic Fluency Test (Spinnler and Tognoni, 1987), the Stroop Test–Short version (Caffarra et al., 2002), and the Wisconsin Card Sorting Test (WCST: Kongs et al., 2000). Last, her perspective-taking capacities abilities were evaluated with Theory of Mind visual stories (ToM1 and ToM2: Amanzio et al., 2008).

Executive functions were also assessed using the Behavioral Assessment of the Dysexecutive Syndrome battery (BADS: Wilson et al., 1996), which is composed of six subtests; each of them evaluates different abilities involved in everyday life, affected by frontal lobe impairment.

Finally, in order to fully assess her executive functions, G.A. performed a Go–NoGo task (Amanzio et al., 2011), which is a response inhibition paradigm, estimating her errors before and after the test.

In line with previous studies (Amanzio et al., 2011, 2016, 2017, 2018; Palermo et al., 2014), we used the Anosognosia Questionnaire for Dementia (AQ-D: Migliorelli et al., 1995), in order to quantify the severity of reduced self-awareness for cognitive and behavioral deficits.

Moreover, a clinician-rated semistructured interview, which is focused on difficulties typically involved in ABI, was applied: the Self-Regulation Skills Interview (SRSI: Ownsworth et al., 2000).

The evaluation of behavioral mood changes was carried out using: the Apathy Evaluation Scale–Clinician version (AES-C: Marin et al., 1991); the Hamilton Anxiety Rating Scale (Hamilton, 1959); the Hamilton Depression Rating Scale (HDR-S: Hamilton, 1960); the Mania Scale (MAS: Bech et al., 1978); and the Disinhibition Scale (Starkstein et al., 2004).

Finally, the functional independence in everyday life was assessed using the Autonomy in Daily Living (ADL: Katz et al., 1963) and the Instrumental Activity of Daily Living (iADL: Lawton and Brody, 1969).

### Results of the Neuropsychological Assessment at T0

Data from the neuropsychological evaluation, concerning the neuropsychological tests and the Go–NoGo task, are listed in Tables 1, 2. The cutoff scores reported in Table 1 (cognitive functions evaluation) are given in the statistical normal direction; the values refer to the normative data for healthy controls matched for age and education.

**TABLE 1:** Neuropsychological assessment: cognitive function evaluation.

	T0 (post-acute phase)		T1 (stabilisation phase)		
	G.A.		G.A.		Cutoff
**Cognitive and intellective assessment**					
MMSE	**18**		**19.3**		≥24
ACE-R	**35**		**41**		≥82
**TIB:**					
Estimated IQ total	**85.306**				90–110
Estimated IQ V	**80.115**				90–110
Estimated IQ P	91.144				90–110
CPM-36	26.2	ES = 3	28.2	ES = 3	≥18.96
**Memory assessment**					
Corsi Block task	4.75	ES = 3	4.75	ES = 3	≥3.5
Digit Span forward	4.25	ES = 2	4.5	ES = 2	≥3.75
Incidental semantic memory	**2**	**ES = 0**	7.01	ES = 1	≥6.67
**Rey memory test**					
Immediate recall	**18.3**	**ES = 0**	**23.1**	**ES = 0**	≥28.53
Delayed recall	**0.7**	**ES = 0**	**2.6**	**ES = 0**	≥4.69
Babcock	**3.75**	**ES = 0**	**0.75**	**ES = 0**	≥7.5
**Language assessment**					
Token test	33.25	ES = 4	33.75	ES = 4	≥26.50
Semantic verbal fluency	**5.5**	**ES = 0**	10.5	ES = 1	≥7.25
**Visuospatial and visuomotor assessment**					
Bells test total score	**24**		**26**		≥32
Bell test (right minus left)	0		0		≤3
Lines bisection	17		17		17
Lines cancellation	40		40		40
Entangled figures test	**6**		**12**		50
**Praxia assessment**					
**Coping design:**					
Without programming elements	8.6	ES = 3	9.1	ES = 3	≥7.18
With programming elements	63.3	ES = 1	65.5	ES = 2	≥61.85
Constructive praxia	10.75		12.75		≥10.25
Ideomotor praxia	19.25		20.25		≥18.42
Buccofacial praxia	**12.25**		20.25		≥18.78
**Perspective taking assessment**					
ToM-1st type	**2**		3		≥3
Comprehension	2		3		
Memory	2		3		
ToM-2nd type	**0**		**2**		≥3
Comprehension	2		2		
Memory	2		2		
**Attentional assessment**					
Attentional matrices	**14.75**	**ES = 0**	**14.75**	**ES = 0**	≥31
TMT A	**689**	**ES = 0**	**650**	**ES = 0**	≤94
TMT B	**NE**	**ES = 0**	**NE**	**ES = 0**	≤283
TMT B-A	**NE**	**ES = 0**	**NE**	**ES = 0**	≤187
**Executive functions assessment**					
MoCA	**16**		**18**		≥26
Phonemic verbal fluency	**11.4**	**ES = 0**	**14.4**	**ES = 0**	≥17.35
Stroop Test—short version:					
Time interference effect	**92.25**	**ES = 0**	**81.25**	**ES = 0**	≤36.91
Error interference effect	**11.5**	**ES = 0**	**14**	**ES = 0**	≤4.24
BADS	**3**		**6**		≥**13**
RSC	0		0		
KS	0		1		
AP	1		2		
TJ	1		2		
ZM	1		1		
MSE	0		0		
**WCST**					
Completed categories	**0**		**2**		≥3
% Errors	**86**		**73**		≤29.90
% Perseverative errors	40		42.478		≤42.70

**Wherever possible Equivalent Scores (ESs) are shown: ES equal to 0 corresponds to a performance of less than 5% of the normal population, thus having pathological meaning. ES equal to 1 indicates a performance to the lower limit of the norm, ES equal to 2 indicates a performance in the standard, ES equal to 3 indicates a performance higher than normal and, finally, ES equal to 4 provides a considerably higher than normal performance. Wherever there is a normative value, the cutoff scores are given in the statistical normal direction; the values refer to the normative data for healthy controls matched for age and education. Abnormal scores are reported in bold. NE, not executable.**

**TABLE 2:** Neuropsychological assessment—part 2: Braver’s Go–NoGo response–inhibition test.

	G.A.		
	T0 (post-acute phase)	T1 (stabilisation phase)	Normal control subjects
**Response inhibition task Go**			
% Target	**70**	**78.8**	98.9
RT (ms)	**467.53**	**396.25**	367
% Errors	**30**	**21.2**	1.1
**Response inhibition task NoGO**			
% Target	**36.4**	**39.6**	85.3
% Errors	**63.6**	**60.4**	14.7
Pre-performance judgment	4/40 X	5/40 X	
Post-performance judgment	5/40 X	4/40 X	

**Comparison with normative data for normal control subjects (Braver et al., 2001). RT, reaction time. Pre-performance and post-performance judgments refer to the number of errors G.A. thought she would do and she did in her opinion. Abnormal scores are reported in bold.**

The neuropsychological screening assessment revealed a global cognitive impairment (MMSE, ACE-R) starting from a premorbid intellectual level slightly below the lower limits of the standard statistical norm (TIB). Moreover, the management of basic and instrumental activities of daily living was compromised. The second assessment phase (cognitive profile completion) revealed difficulties in the following functions (values below the cutoff): visual perception (Bells Test) and visual recognition of complex percepts (test of the entangled figures), buccofacial praxia, memory (Babcock, Rey Memory Test), learning (Incidental Semantic Memory Test), access to the internal vocabulary (semantic fluencies), and first and second type of Theory of Mind. A loss of attentional capabilities was also found (Attentional Matrices, TMT-A). The MoCA score revealed the presence of executive dysfunctions, confirmed by BADS, TMT-B, phonemic fluency, Stroop test, and WCST in terms of cognitive set-shifting, inhibition of dominant responses, flexible thinking, and monitoring.

As to the performance obtained on the response inhibition test, G.A. made four times the number of errors made by healthy controls in the NoGo condition. Nevertheless, she was not able to predict the number of her errors or to monitor her own performance.

Data from the neuropsychiatric, functional, and self-awareness evaluation are listed in Table 3. At T0, the evaluation detected a mood deflection under cutoff in terms of depression and apathy. It is noteworthy that AQ-D scores indicated a great impairment in self-awareness, AQ-D iADL, and AQ-D depression domains. G.A. judged the implications of the pathological event as less severe than its real nature, especially when compared with the perception of the primary caregiver.

**TABLE 3:** Neuropsychological assessment—part 3: awareness of deficits, neuropsychiatric, and functional assessment.

	T0 (post-acute phase)			T1 (stabilisation phase)			
	Caregiver	G.A.	Total score	Caregiver	G.A.	Total score	Cutoff
**Awareness assessment**							
AQ-D overall	62	19	**43**	47	11	**36**	≤14
AQ-D cognitive part	46	15	31	38	7	31	
AQ-D behavioral part	16	4	12	8	3	5	
AQ-D ADL	4	2	2	2	2	0	≤4
AQ-D iADL	28	11	**17**	24	5	**19**	≤4
AQ-D depression	14	6	**8**	12	6	**6**	≤4
AQ-D disinhibition	0	0	0	0	0	0	≤4
SRSI						28	
**Neuropsychiatric assessment**							
AES-C			**27**			**29**	≥37.5
HDR-S			**11**			**9**	≤7
HAR-S			9			8	≤17
Disinhibition scale overall			15			14	≤16.9
Apathy			9			8	
Abnormal motor behavior			1			1	
Stereotypy			1			1	
Hypomania			1			1	
Psychosis			1			1	
Poor self-care			2			2	
MAS			4			3	≤15
**Functional assessment**							
ADL			**1**			6	6
iADL			**2**			**3**	5

*Note: abnormal scores are reported in bold.*

### Results of the Neuropsychological Assessment After 6-Month Follow-Up (T1)

G.A. underwent a new neuropsychological assessment, using the same batteries performed at T0 (Tables 1–3). The patient seemed to show some improvements in her cognitive functioning but, unfortunately, she still exhibited several cognitive deficits. A specific and persistent reduced self-awareness associated with executive dysfunction and mood changes in terms of apathy and depression were also still present.

Although G.A. showed improvements in terms of overall evaluation, she still exhibited several cognitive deficits considering: visual perception (Bells Test) and visual recognition of complex percepts (test of the entangled figures), and memory (Babcock, Rey Memory Test). Attentional deficits (Attentional Matrices, TMT-A) and executive dysfunction (BADS, TMT-B, Stroop test, WCST and Go–NoGo task) were still present. Moreover, she exhibited a persistent apathetic mood orientation (AES-C).

Indeed, G.A. was still unaware of her cognitive and behavioral deficits (in terms of global AQ-D, AQ-D iADL, and AQ-D depression). Moreover, SRSI revealed that G.A. was unable to self-monitor everyday activities, to anticipate outcomes and consequences, or to adopt new strategies in order to better face events. Awareness of functional implications, expectations of recovery, and need for treatment were not sufficient at the time of evaluation.

### Results of the Serology Test in Chemiluminescence (CLIA) to Detect IgG Antibodies Against SARS-CoV-2

The serology test, carried out on May 18th, 2020, highlighted IgG antibodies present/IgM absent: high probability of previous contact with the virus (at least prior to 20–25 days).

This is an indirect, quantitative test, approved by the Italian Ministry of Health (Ministero della Salute, 2020), which highlights the immune system’s response to infection following contact with the coronavirus.

## Discussion

This case report can contribute to novel insights about the neuropsychological consequences of ABI (Rusconi et al., 2014). Importantly, G.A. was affected by one of the rare cases of bilateral thalamic infarction. In fact, as documented by Kumral et al. (2001), over 2,750 patients with ischemic stroke, only 0.6% of them present this kind of infarction.

From an etiopathogenetic point of view, G.A.’s bilateral thalamic lesion was conceivably produced by an unfortunate combination of paramedian and polar artery territories infarction (Perren et al., 2005).

At T0, the neuropsychological evaluation underlined a cognitive impairment due to ABI. She showed deficits in visual perception and visual recognition of complex percepts, buccofacial praxia, attention, memory, access to the internal vocabulary, and first and second type of Theory of Mind. Executive dysfunction, in terms of cognitive set-shifting, flexible thinking, inhibition of dominant responses, and monitoring, were also present. She also showed functional disorders and mood changes, in terms of depression and apathy. Finally, she presented a clear-cut reduction in self-awareness.

Six months later, during the follow-up phase (T1), cognitive, behavioral, and functional disorders persisted. Cognitive deficits, in terms of visual perception and visual recognition, attention, and memory, were still present.

It is noteworthy that impaired self-awareness and executive dysfunctions, concerning response-inhibition, cognitive set-shifting, and monitoring abilities, were present as residual deficits after the bilateral thalamic infarction in the post-acute phase. Importantly, the secondary disruption of self-awareness related to executive dysfunction might arise from frontal impairment or from frontal-subcortical damages (O’Keeffe et al., 2007). Considering that frontal-subcortical or diencephalic structures injuries could be predisposing factors to produce reduced self-awareness, reduction of self-awareness may be a direct consequence of frontal and diencephalic damages after lesions. This mechanism could clarify the reason why patients with a right temporoparietal or thalamic damage do not always present these clinical deficits (Starkstein et al., 1992). It is important to point out that ABI was bilateral in G.A.: in this case, it should be hypothesised a more severe clinical picture related to reduced self-awareness. Moreover, the findings by Starkstein et al. (1992) supported Nielsen’s (1938) suggestion that unawareness of deficits “may be caused by thalamic lesions or isolation of the thalamus from the parietal cortex” (Starkstein et al., 1992, p. 1,452).

Consistent with the findings in G.A., O’Keeffe et al. (2007) suggested that any deficits in monitoring, response inhibition or cognitive flexibility, can affect patients’ foresight (i.e., self-awareness).

Thus, probably G.A.’s impairment in the maintenance of cognitive representation of her experimental tasks through self-monitoring—determined by control executive processes sustained by the connection between dorsolateral and medial prefrontal cortices—may have a function in her reduced self-awareness (Palermo et al., 2014).

While considering the different etiopathogenesis between ABI and neurodegenerative disorders, the similarity of the neuropsychological profile associated with the onset of reduced self-awareness seems to authorise a transposition of the interpretative models. Therefore, we can affirm that overlapping symptoms appear consequently to specific dysfunction of the frontal networks and of the diencephalic structures. In fact, deficits in response inhibition, set-shifting, and self-monitoring contribute to reduced self-awareness (Amanzio et al., 2011, 2013, 2014, 2016; Palermo et al., 2014, 2015, 2017).

Considering the relationship between subcortical area and awareness, Ownsworth et al. (2008) reported how thalamic damages seemed to generate various deficits in functional domains, such as cognitive, motor, sensory, and perceptual processes (Kumral et al., 1995). Instead, mood changes and reduced self-awareness can be observed with right thalamic injuries (Leibson, 2000). Finally, direct lesion of the thalamus and disconnection of different cortical networks (for example, frontal-thalamic pathways) were related to impaired awareness and emotional dysregulation (Bogousslavsky, 1994). However, these studies have the limitation of considering heterogeneous lesion sites and, consequently, patients with dissimilar cognitive disorders as unicum.

As stated above, mood changes observed in G.A. are not surprising. She obtained abnormal, but near the cutoff, scores on HDR-S and AES, in both T0 and T1. These results suggest that G.A. had a slight change in behavior, even if not so psychopathologically relevant.

G.A.’s executive dysfunction—together with the Cognitive Awareness Model (CAM), a neurocognitive model proposed by Agnew and Morris (1998)—help to comprehend the impact of executive system on self-awareness in ABI patients (Agnew and Morris, 1998). Consciousness of changes following injuries needs an interaction among comparator mechanisms of the executive system (in order to perceive modifications), different functional domains (such as cognitive, motor, sensory, and perceptual ones), and the metacognitive awareness system. Being characterised by a feedback mechanism, the CAM depends on updated information about experiences of success or failure. Consequently, it is important for self-knowledge on personal capacities and deficits (Morris and Hannesdottir, 2004).

The present study seems to suggest that G.A.’s comparator mechanisms, responsible for monitoring attentive performance, were compromised; however, the unfortunate co-occurrence of thalamic bilateral injury cannot be considered as the only explanatory mechanism.

Nevertheless, G.A.’s comparator mechanisms become unable to detect discrepancies between actual and past performances, failing in cognitive tasks both of everyday activities and during test sessions.

These neuropsychological changes can make the patient less able to understand risky situations and affect the iADL. Such aspects may promote exposure to infection, as the patient may not be able to take preventive measures (e.g., social distancing, hand washing, and face mask use). The latter could represent only a suggested hypothesis, which needs to be verified through in-depth multidisciplinary investigations (neuropsychological, virological, and epidemiological).

Finally, it is important to underline that a recent article (Kummer et al., 2020) showed how a history of stroke was significantly related to poor prognosis among SARS-CoV-2 patients, admitted in the hospital from March to May 2020. Considering these results, which further studies should confirm, the authors suggested how patients with COVID-19 have higher probabilities of poor illness outcomes (Kummer et al., 2020).

## Limitation Section

Although our results were collected from a single case study, and therefore cannot be generalised to the entire population, our work is based on strong neuropsychological methodologies that allow us to substantiate our inferences. However, future studies are needed in order to understand how metacognitive–executive dysfunction and reduced awareness can influence the possible risk of SARS-CoV-2 contagion in the elderly population.

## Conclusion

This case study contributes to the poor literature on the impact of a bilateral thalamic lesion on self-awareness. Future studies will help to better analyse the possible impact of bilateral frontothalamic lesions in subjects with a reduction of self-awareness and executive dysfunction, through the combined results of MRI and positron emission tomography, in order to document a possible framework of atrophy and hypometabolism at the level of the medial prefrontal cortex.

We believe that studying reduced self-awareness more closely may be useful for developing compliance to the treatment of COVID-19 patients. Currently, older people in Italy represent the category at greatest risk, as reported by the epidemiological analysis of infected and deceased subjects due to COVID-19 (Ministero della Salute, 2020). Executive dysfunction, which makes the supervision of the iADL inadequate (Amanzio et al., 2016, 2017, 2018), could lead to a greater risk of contagion in elderly patients with neurological disorders.

Considering the above, a supervision of the iADL and rehabilitation programs of executive dysfunction might be somehow helpful in reducing the impact of COVID-19, making elderly less susceptible to the contagion.

## Ethics Statement

The study was approved by the Ethics Committee “A.O.U. Cittàă della Salute e della Scienza di Torino – A.O. Ordine Mauriziano – A.S.L. Cittàă di Torino”İ as part of the core research criteria followed by the Neurological Unit. All the implemented procedures ensured the safety, integrity, and privacy of the patient. The primary caregiver had previously read the information sheet about the patient rights and signed the informed consent for the use of GA personal data for scientific purposes and research. Any critical aspects, neither with regard to the MRI acquisition nor to the neuropsychological assessment, could be noticed. Importantly, the study has been conducted according to the principles set forth by the Declaration of Helsinki (59th WMA General Assembly, Seoul, October 2008) and in accordance with the Medical Research Involving Human Subjects Act (WMO).

## Author Contributions

MB and SP collected the neuropsychological data. MB, SP, and MA contributed to the conception of the study and wrote the first draft of the manuscript. DL performed the neurological evaluation. MS and MV made the neuroradiological diagnosis. MB, SP, and GEC wrote the second version of the manuscript. MA revised the manuscript critically. All authors contributed to the article and approved the submitted version.

## Conflict of Interest

The authors declare that the research was conducted in the absence of any commercial or financial relationships that could be construed as a potential conflict of interest.
